# Neurobiological Causal Models of Language Processing

**DOI:** 10.1162/nol_a_00133

**Published:** 2024-04-01

**Authors:** Hartmut Fitz, Peter Hagoort, Karl Magnus Petersson

**Affiliations:** Donders Institute for Brain, Cognition and Behaviour, Radboud University, Nijmegen, The Netherlands; Neurobiology of Language Department, Max Planck Institute for Psycholinguistics, Nijmegen, The Netherlands; Faculty of Medicine and Biomedical Sciences, University of Algarve, Faro, Portugal

**Keywords:** adaptive dynamical systems, computational modeling, implementational causality, neurobiology of language, processing memory

## Abstract

The language faculty is physically realized in the neurobiological infrastructure of the human brain. Despite significant efforts, an integrated understanding of this system remains a formidable challenge. What is missing from most theoretical accounts is a specification of the neural mechanisms that implement language function. Computational models that have been put forward generally lack an explicit neurobiological foundation. We propose a neurobiologically informed causal modeling approach which offers a framework for how to bridge this gap. A neurobiological causal model is a mechanistic description of language processing that is grounded in, and constrained by, the characteristics of the neurobiological substrate. It intends to model the generators of language behavior at the level of implementational causality. We describe key features and neurobiological component parts from which causal models can be built and provide guidelines on how to implement them in model simulations. Then we outline how this approach can shed new light on the core computational machinery for language, the long-term storage of words in the mental lexicon and combinatorial processing in sentence comprehension. In contrast to cognitive theories of behavior, causal models are formulated in the “machine language” of neurobiology which is universal to human cognition. We argue that neurobiological causal modeling should be pursued in addition to existing approaches. Eventually, this approach will allow us to develop an explicit computational neurobiology of language.

“You can’t go to a physics conference and say: I’ve got a great theory. It accounts for everything and is so simple it can be captured in two words: *Anything goes*.” (Noam Chomsky, 2022, as cited in Marcus, 2022)

## THE CORE COMPUTATIONAL MACHINERY FOR LANGUAGE

Sentence comprehension requires at least two functional components, a long-term storage of words and their feature structure (mental lexicon) and a combinatorial device (unification) that integrates sequential information into structured representations over time (Hagoort, 2005, 2019; Jackendoff, 2002). These components interact during real-time incremental processing and mutually control each other. This process involves linguistic representations at different grain sizes, from phonemes to words, phrases and sentences (Dehaene et al., 2015), and memory on multiple time scales, ranging from milliseconds to minutes and a lifetime (Hasson et al., 2015). An adaptive processing dynamics shaped by ontogenetic development (genes and experience) operates on these linguistic primitives and ties them together in processing memory (Petersson & Hagoort, 2012). The computational machinery that supports these operations is implemented in neurobiological infrastructure at different spatial scales, from single neurons and synapses to cortical layers, microcolumns, brain regions and large-scale networks. A theory of language processing that aims to be *complete* needs to explain how this machinery is realized within the neurobiology of the language system across spatial and temporal scales. (We use “language system” as short-hand for language-relevant brain regions without implying that these regions are functionally exclusive to language.) This explanatory goal is shared by most researchers in the field, but an integrated account has not been accomplished thus far. Some have argued that we lack even the most basic understanding of how linguistic units are represented and stored in long-term memory (Poeppel & Idsardi, 2022). In a similar vein, the neurobiological basis of processing memory for unification is currently unknown (Fields, 2022; Fitz et al., 2020). In this perspective article, we describe a computational modeling approach that maps out a way forward for the language sciences in order to achieve this explanatory goal. This approach aims toward a fundamental understanding of core language function from first principles of neurobiology.

## MULTIPLE EXPLANATORY STRATEGIES

Language as a neurobiological system needs to be distinguished from its behavioral output, which includes speech, sign, or text in production and sentence interpretations in comprehension. Although the language system is used for communication and thinking, these phenomena should not be mistaken for the system itself (Jackendoff, 2002). A key question is how to link behavioral output to the computational machinery of the neurobiological system that generates the output. This is one of the fundamental challenges in explaining natural language in mechanistic terms.

**Figure 1.**
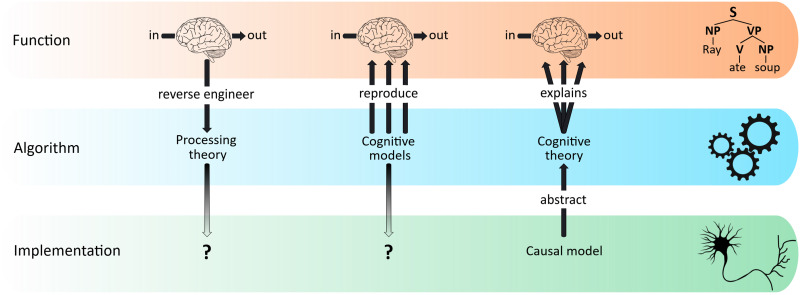
Three approaches toward understanding language as a cognitive system. Cognitive information processing systems can be described at different levels of explanation, here exemplified by the functional, algorithmic, and implementational levels (Marr, 1982). A complete understanding of such a system would allow us to traverse seamlessly between levels in all directions. Although the three levels will have to be augmented with additional ones (Churchland & Sejnowski, 1988; Tanenbaum & Austin, 2013), this broad distinction has been fruitful in partitioning the problem space. This explanatory challenge can be approached in different ways. Experimental language science has attempted to infer processing theories from observed input–output relations (left). Cognitive modeling has proposed a large array of algorithms that can each reproduce some aspects of these relations (center). Causal modeling starts from neurobiological principles to synthesize an explanatory language model which is, first and foremost, a model of the system itself (right). Ideally, such a model will eventually explain all behavioral data generated by the system.

The experimental approach sets out at the top or functional level of description and attempts to infer processing theories from measured input-output relations (Figure 1). (Note, the top level refers to *what* is being computed, i.e., which recursive function *ϕ*. Marr’s [1982] term “computational level” is unfortunate because it creates confusion with algorithm.) These are often informal verbal theories that do not reach algorithmic specificity. Moreover, current experimental methods are relatively coarse and do not allow the reconstruction of simple computational devices whose functionality is known (Jonas & Kording, 2017). This complicates the reverse engineering of cognitive systems from experimental data, which therefore has to be complemented with other methods. One such approach has tried to map these relations algorithmically through cognitive modeling. Different frameworks have been proposed (e.g., connectionist, symbolic, hybrid, Bayesian) that each captures some aspect of language behavior, but so far this approach has not resulted in a unified picture of linguistic computation. Since any finite collection of data can be re-coded by many different formalisms, success in approximating behavior algorithmically does not automatically guarantee neurobiological realism. The chances that a stipulated algorithm provides a correct description of the actual computational machinery is small, no matter how well the formalism fits with behavioral data. Independent evidence is needed to establish realism which, by necessity, must stem from the neurobiological characteristics of the very system that is being modeled. In the absence of such neurobiological constraints, cognitive models remain high-level abstractions whose relationship to the implementational substrate is unclear.

**Figure 2.**
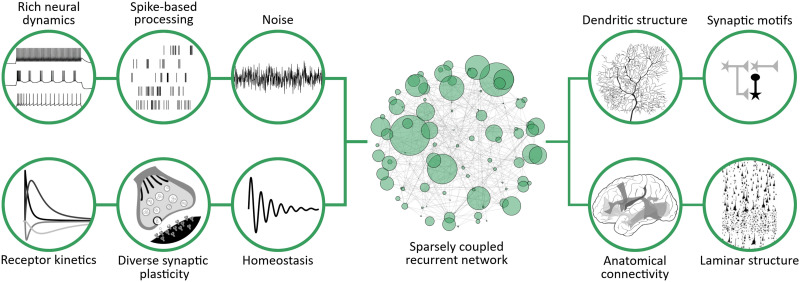
First principles of neurobiology. Features of the nervous system that are largely uncontroversial in neurobiology form the basis of causal language models. These can be viewed as boundary conditions that constrain proposed mechanisms for language processing. Causal modeling seeks to understand the computational role of these features in relation to language processing and integrate the implementational level with the algorithmic and functional levels of description.

For these reasons, we argue that a third explanatory strategy should be pursued urgently, and concurrent with, the more traditional approaches shown in Figure 1. This strategy puts a premium on neurobiology as a primary source of evidence and attempts to model the language system at the implementational level of description. We refer to this approach as *neurobiological causal modeling*. In our terminology, a causal model is a set of functional equations that describes the dynamics of a system at the level of neurobiological causality. This concept differs from the structural causal models of Pearl (2000), dynamic causal modeling (Friston et al., 2003), or models of causality itself (Granger, 1969). A causal model is built directly on established neurobiological principles without making ad hoc assumptions about algorithmic procedures and component parts (Figure 2). The goal of this approach is to synthesize an explanatory language model that can uniformly explain linguistic behavior across different experiments. Unlike most existing approaches, causal modeling draws on a wealth of additional insights from neuroanatomy (Petrides, 2013; Tremblay & Dick, 2016), neurophysiology (Kandel et al., 2012; Luo, 2015; Sterling & Laughlin, 2015), and biophysics (Koch, 1998) that inform model construction. The implementational building blocks derived from these knowledge sources can provide the necessary constraints for a computational neurobiology of language that ultimately integrates across all levels of description.

The case for neurobiological constraints on models of cognition was made in the seminal work of Churchland and Sejnowski (1992) and has been reiterated by others since then (e.g., Astle et al., 2023; O’Reilly, 2006; Pulvermüller et al., 2021). One way to approach this issue is to constrain existing neurocognitive architectures in order to increase their biological plausibility (Pulvermüller et al., 2021). Another approach, which we advocate here, is to systematically assemble computational language models from known neurobiological primitives at the implementational level (Figure 2). Although superficially similar, the former approach is reductive in nature while the latter is synthetic. To prevent early misunderstanding, neurobiological causal language modeling does not strive to dispense with function or algorithm, which are integral to a complete explanation. On the contrary, causal modeling aims to firmly ground linguistic behavior and cognitive theory in the causal characteristics of the actual language system and its concrete neurobiological instantiation.

## FIRST PRINCIPLES OF NEUROBIOLOGY

The language system of the human brain is a particular instance of a sparsely connected recurrent network of biological neurons and chemical synapses. This theoretical framework is sufficiently expressive to capture *all* anatomical connectivity, including connectivity between brain regions, the laminar structure within cortical columns, synaptic motifs within and between layers, and randomness at the microscopic scale. In the context of recurrent networks, there is no fundamental difference between connectivity patterns at different spatial scales. Since a static structured connectome by itself is nonexplanatory (Bargmann, 2012), it is critical to also realistically model neural interactions and the information flow across this graph.

Fast signaling in the nervous system is based on action potentials, which are all-or-none neuronal responses to analog input. Spikes are the basic units of cortical information processing, and it has been argued that their temporal relations play an important role in the encoding, representation, and transmission of processing outcomes (Brette, 2015; Gerstner et al., 1997). Neurobiological language models are needed that can express the temporal dimension of spike-based processing and resolve the mismatch between the time scales of action potentials and cognitive behavior (Chaudhuri & Fiete, 2016). Biological neurons exhibit a wide range of electrophysiological behavior, from tonic spiking to bursting and adaptation, and this diversity of observed firing patterns is likely to have functional significance (Gerstner et al., 2014; Koch, 1998). Neuronal spike responses result from the integration of synaptic inputs on the spatial structure of the dendritic tree, which amounts to more than linear summation. The spatio-temporal nature of dendritic integration gives rise to complex, nonlinear processing effects that are not captured by simpler point neurons (Gidon et al., 2020; London & Häusser, 2005; Payeur et al., 2019). Thus, the input–output behavior of neurons as the fundamental computational unit is substantially richer than has been assumed (Larkum, 2022). Dendritic morphology is one of the candidate features that may account for species-specific cognitive functions (Fişek & Häusser, 2020), including language, and multicompartment neuron models can be viewed as interconnected computational elements that are all potential targets for learning and adaptation (see Quaresima et al., 2023, for an explicit modeling account).

Neurons connect via excitatory or inhibitory synapses but not both at the same time, and synapses do not change sign during learning and development (Strata & Harvey, 1999), as is the case in virtually all connectionist and deep learning models of language processing. Major synapse types include fast and slow excitatory and inhibitory ones that generate postsynaptic currents with different polarity, amplitudes, and rise and decay time scales (Destexhe et al., 1998). Synaptic learning and memory are subserved by a variety of unsupervised learning principles (Magee & Grienberger, 2020) that include activity-dependent, short-term synaptic changes (Markram et al., 1998), mechanisms for long-term potentiation and depression based on the timing of pre- and post-synaptic spikes (Markram et al., 1997), as well as synaptic consolidation on much longer time scales (Clopath, 2012). In addition, reward-modulated learning (Frémaux & Gerstner, 2016) and more powerful error-driven learning mechanisms also play a role (Payeur et al., 2021; Whittington & Bogacz, 2019).

To temper runaway processes due to Hebbian plasticity, homeostatic mechanisms need to ensure that single-neuron and circuit firing rates remain within physiological ranges (Tetzlaff et al., 2012; Turrigiano & Nelson, 2004). These mechanisms act, e.g., by scaling synaptic conductances or by downregulating neuronal excitability. Furthermore, language-relevant networks need to function in the presence of endogenous background activity and stochastic variability at the cellular and synaptic level (Faisal et al., 2008; Nolte et al., 2019). These noise sources reduce the computational capacity of the system to that of Turing machines with finite tapes, i.e., finite-state machines, by limiting processing precision and effective memory capacity (Maass & Orponen, 1998; Petersson, 2005). In addition, we note that in parallel with the fast processing systems outlined above, there are neuromodulatory systems (e.g., monoamines, neuropeptides) that are different in nature from the fast conductance-based signaling systems. They typically originate in the midbrain/brainstem, with widespread cortical–subcortical projections, operate on longer time scales, and directly regulate the intracellular biochemistry via G protein coupled receptors. These systems modulate fast neural processing, and it has been suggested that they support unconventional computation and neuronal memory (Bechtel, 2022; Bray, 2009; Koch, 1998).

This inventory of neurobiological principles constitutes the foundation of causal modeling and imposes strong constraints on the computational realization of language (Figure 2). Importantly, these constraints are both constructive and limitative. On the one hand, they specify the basic building blocks of neurobiological language models and thus provide an evidence-based implementational scaffold for causal modeling. Mathematical models of these component parts have been carefully developed by experimental and theoretical neuroscientists to closely capture the net effects of physiological processes quantitatively (Box 1). The objective of causal modeling is to explain language processing in terms of these neurobiological principles that characterize the mechanics of the real system. On the other hand, these constraints curb arbitrary choices made in cognitive language modeling and deep learning models at the level of component parts and algorithms. In order to establish valid abstractions, it is necessary to scientifically demonstrate that these abstractions can be reduced to the level of neurobiological implementation. Pending such reductions, algorithmic explanations that are obtained by abstracting away from elementary features of the nervous system run a high risk of being spurious.

Box 1. Causal modeling toolbox.
Although causal models operate at the implementational level, the aim is not to replicate reality in all its complexity. Instead, physiological processes are modeled in a phenomenologically effective manner. For many of the neurobiological features in Figure 2, reduced mathematical models exist from which causal networks of language function can be assembled, largely in the form of systems of coupled differential equations.

The distribution of cortical spikes can, under suitable circumstances, be approximated by Poisson processes (Softky & Koch, 1993) to encode input as frozen noise. This is an example of how one can create a spatio-temporal code for linguistic units which carries more information than a rate-based code (Duarte et al., 2018; Uhlmann, 2020). The two-dimensional, adaptive-exponential neuron is able to produce a wide range of firing patterns (Brette & Gerstner, 2005) and accurately predicts in vitro spike times (Rossant et al., 2011). Synapses can be modeled as alpha functions or the difference between two exponentials that describe the rise and decay times of post-synaptic currents (Roth & van Rossum, 2009), and conductance-based coupling supports realistic population dynamics (Cavallari et al., 2014). Event-driven simulation can be used to efficiently model axonal delays for long-range connectivity patterns. Short-term synaptic facilitation and depression is modeled in terms of neurotransmitter release probability and depletion (Markram et al., 1998), and this mechanism has been implicated in working memory function (Mongillo et al., 2008). Excitatory long-term potentiation and depression are conceptualized as Hebbian spike-timing dependent plasticity (STDP). Several similar formalisms exist which are based, e.g., on triplets of spikes (Pfister & Gerstner, 2006), or on pre- and post-synaptic voltage traces (Clopath et al., 2010). The latter rule allows for strong bidirectional potentiation, which has been observed experimentally. To counteract dynamic instability due to STDP, inhibitory plasticity acts on inhibitory synapses to maintain a target firing rate (Luz & Shamir, 2012; Vogels et al., 2011). This form of plasticity also establishes a local balance between excitatory and inhibitory synaptic inputs to each neuron and is conducive to achieving asynchronous, irregular spiking activity, which plays an important role in cortical information processing (Herstel & Wieringa, 2021; van Vreeswijk & Sompolinsky, 1996). Synaptic normalization is another homeostatic principle which counteracts uncontrolled synaptic growth due to STDP while preserving synaptic specificity (Turrigiano, 2008). On longer time scales, relevance signaling and synaptic tagging models have been developed that prevent overwriting and enable memory consolidation (Clopath et al., 2008; Ding et al., 2022; Ziegler et al., 2015). What has been missing from this inventory of neurobiological components, until recently, are computationally efficient multicompartmental neuron models, capable of reproducing nonlinear dendritic integration effects that have been described experimentally (Koch, 1998; London & Häusser, 2005; Payeur et al., 2019; Poirazi & Papoutsi, 2020). The Tripod neuron proposes a structural reduction of the dendritic tree to fill this gap and can now be used to investigate the functional role of dendritic integration in large networks (Quaresima et al., 2023).

Causal language modeling is further supported by flexible, high-level spiking network simulators (Gewaltig & Diesmann, 2007; Stimberg et al., 2019), code-sharing platforms (McDougal et al., 2017), and programming languages for high performance scientific computing (Bezanson et al., 2017).

## DYNAMICAL SYSTEMS VIEW ON LANGUAGE

**Figure 3.**
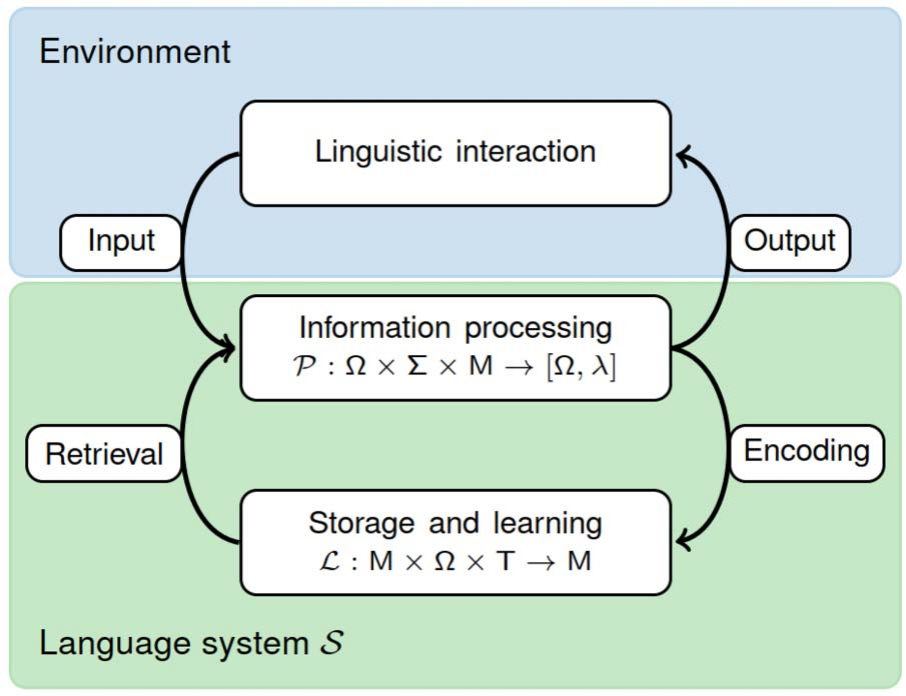
Schematic of an adaptive information processing system 𝒮 for language. Based on input from the system embedding (environment; an element of Σ), the current state (an element of Ω), and current parameters in the model space M, the processing dynamics 𝒫 traces out a trajectory in neural state space and returns language output *λ*. The learning mechanisms 𝓛 are coupled to 𝒫 creating continuous cycles of information encoding into, and retrieval from, memory that operates on multiple time scales for short-term and long-term storage as well as development. For instance, in the case of ontogenesis, 𝓛 implements developmental processes and genetically guided maturation dependent on time T, while 𝒫 instantiates the parsing capacity that evolves towards adult competence as a function of 𝓛’s trajectory through the model space M. On shorter time scales, 𝓛 implements an active processing memory, and because the form of 𝓛 is structurally similar to 𝒫, it is possible that learning and memory mechanisms are actively computing on relevant time scales as well (e.g., in transforming episodic memories into general world knowledge as a consequence of repetition during consolidation). Figure adapted from Petersson and Hagoort (2012).

The neurobiology of language fits naturally within a description of language processing in terms of a specific continuous-time adaptive dynamical system built from neurobiological components. Here we provide a terse mathematical formalization of such a system 𝒮 in terms of interacting functional components that are coupled via a neurobiologically specified processing dynamics 𝒫 and adaptive learning mechanisms 𝓛 (Figure 3). Note that 𝒫 and 𝓛 are multivariate and each component is associated with a physical measurement unit.

𝒫 maps an internal state *s* ∈ Ω and an input *i* ∈ Σ onto a new internal state $\hat{s}$ = *s*(*t* + *dt*) = *s*(*t*) + *ds*(*t*). States *s* are real-valued tuples of dynamical variables in neurobiology, e.g., membrane potentials and synaptic conductances, that describe the language system across all spatial scales. Input *i* is provided to 𝒫 by the environment that the system is embedded through an interface (e.g., a speech sound transduced by the cochlea), and the optional output *λ* of 𝒫 is translated into an internal action or an external motor response (e.g., articulation). State transition is characterized by coupled stochastic differential equations *ds*(*t*) = 𝒫(*s*, *i*, *m*)*dt* + *dξ*(*t*) that are parameterized in *m* (see below) and coupled to noise processes *ξ*(*t*). Thus, information processing is represented as an input-driven, or *forced*, trajectory through the system’s state space Ω and, importantly, is constrained by the dynamics 𝒫. In classical terminology this is the infinitesimal version of the process logic of a Turing machine, i.e., its machine or transition table. For instance, in language comprehension, a subsystem of 𝒫 can be understood as the parser associated with 𝒮. Since *ds*(*t*), and therefore the next state $\hat{s}$, is recursively determined by the continuous action of 𝒫, language processing in this framework is naturally incremental, recursive, and state-dependent, as in classical theories of computation (Buonomano & Maass, 2009; Petersson & Hagoort, 2012).

𝒫 is intertwined with a dynamics 𝓛 for development, learning, and adaptation that governs the evolution of 𝒮 as a function of linguistic experience and maturation. This is formalized as *dm*(*t*) = 𝓛(*m*, *s*, *t*)*dt* + *dη*(*t*) where the learning parameters *m* belong to the model space M = {*m*|*m* can be realized by 𝒮} and *η*(*t*) is another noise process. The elements of M are high-dimensional tuples of synaptic, neuronal, and other adaptive parameters in the language network, and the dynamics 𝓛 is a set of neurobiological learning principles. In contrast to 𝒫, 𝓛 is explicitly dependent on time T which captures the notion of innately guided maturation processes. At any point in time, 𝒮 is in a particular developmental state *m*(*t*) and 𝓛 carves out a trajectory in M as the system matures. However, since 𝓛 is coupled back to 𝒫 via *m*, the processing characteristics of 𝒮 themselves change over time, and the fixed points of 𝓛 mark the developmental end-state of adult competence. Prior knowledge of language (Chomsky, 1986) is incorporated into 𝒮 as a structured initial state *m*(*t*_0_), or as additional constraints on 𝒫, 𝓛, or M, the so-called language acquisition device (cf. Petersson & Hagoort, 2012). The initial state is the outcome of gene-regulatory development of the language-ready brain, optimized by biological evolution, and subsequently fine-tuned through linguistic experience during acquisition (Zador, 2019). Due to the fact that the complete dynamics of 𝒮 is also shaped by linguistic interaction with a cultural environment, the neurobiological language system is a biocultural hybrid (Evans & Levinson, 2009).

Consequently, the general form of the language system 𝒮 is an adaptive system of interacting dynamical variables in neurophysiology whose state transitions are determined by the coupled dynamics for processing 𝒫 and learning/development 𝓛. At any developmental stage, the algorithmic nature of 𝒫 and 𝓛 is determined by neurobiology, and one objective of causal modeling is to characterize these dynamics and interpret them in language processing terms. Without a cognitive interpretation, 𝒮 remains an unanalyzed system that moves in time. Another important goal of causal modeling is to identify the language-relevant representational states of 𝒮 which are expected to be evoked spatio-temporal transients in ongoing processing (Petersson, 2008; Rabinovich et al., 2008).

The dynamical systems perspective characterizes language processing in full generality and with formal precision. This allows us to clearly identify the different *explananda*—processing, learning, maturation, and the initial state—and how they interact. Component parts of causal models are expressed as continuous-time differential equations coupled into a functional architecture defined by the connectome. Every instantiation of a causal language model built from such component parts *ipso facto* is a specific claim about, and a concrete algorithmic proposal of how, the processing and learning dynamics 𝒫 and 𝓛 could be implemented at the level of neurobiology. Hence, there is a natural relationship between the neurobiological dynamical system and causal language modeling, whereas this link is either missing or contrived for models that are not formulated in causal terms.

## HIERARCHY AND BINDING IN NEURAL PROCESSING

Language is characterized in terms of hierarchical structures that describe the representations that the comprehension system needs to compute when parsing an utterance. Hierarchical dependencies between constituents are ubiquitous at all linguistic levels, from phonemes and syllables to words, phrases, clauses, and sentences (Hagoort, 2019; Hasson et al., 2015; Jackendoff, 2002). At the same time, language processing is subserved by recurrent networks of spiking neurons and chemical synapses, and it is not obvious how hierarchical linguistic structure can be mapped to neurobiology. Thus it has been an enduring debate how neural systems can accomplish so-called hierarchical processing and this issue is closely tied to the binding problem.

**Figure 4.**
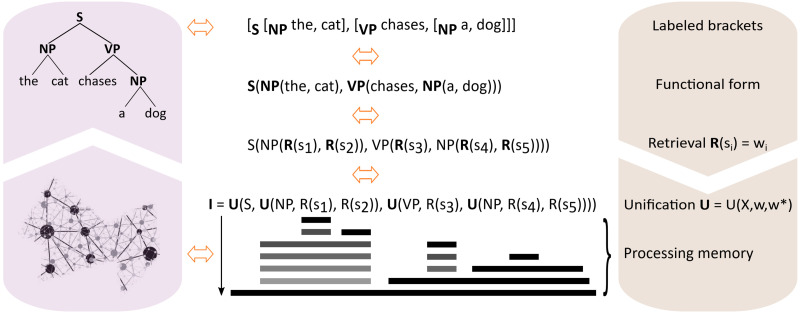
Translating linguistic hierarchy into neural processing. Phrase structure trees are rewritten in labeled bracket notation where brackets correspond to nodes in the tree and labels indicate the category of nodes (orange arrows represent equivalence). Labeled brackets can be expressed functionally as **NP** (“the, cat”), and similarly for other phrasal categories in the example sentence. Words that enter into these function calls are retrieved from the mental lexicon by an operator **R** that incrementally maps speech sounds **s**_*i*_ onto word representations **w**_*i*_. A parameterized function **U** (unification) is introduced that takes three arguments, a phrasal category and two partial interpretations *w*, w* that have either been retrieved by **R** or computed by previous actions of **U**. To establish sentence meaning, nested function calls to **U** are executed in the correct order as soon as relevant information becomes available (immediacy principle) and the output of **U** corresponds to the interpretation **I** of an utterance. During this procedure, lexical items as well as partial interpretations previously computed by **U** have to be kept in processing memory until they are being integrated. Processing memory also keeps track of which components have already been unified, and when, in order to carry out potential revision. Grayscale horizontal bars show the lifetime of information content temporarily held in memory at each processing step, the vertical arrow indicates logical time. NP = noun phrase; VP = verb phrase.

The apparent conflict between these notions can be resolved when static structural hierarchy (represented by parse trees) is interpreted dynamically in neural processing terms (Figure 4) where words are retrieved from the mental lexicon by an operator **R** and unified combinatorially by a universal function **U**. Hierarchical processing corresponds to nested function calls, including recursion, that are executed by the neural parser at the appropriate point in logical time, augmented with a memory structure, or unification space, to store and retrieve intermediate results when needed. The control input for **U** parametrically switches unification into different subroutines by function composition. It is supplied by the feature structure of retrieved words (e.g., lexical categories) or computed internally within processing memory from the available information (e.g., phrasal categories). Biological networks for unification thus require distinct input lines for data and control, similar to the pins on a microprocessor. In neurobiology this can be achieved by electrotonically segregated dendritic branches that integrate different input types independently (Larkum, 2022; Spratling, 2002). For example, basal and apical dendrites of cortical pyramidal neurons receive inputs from anatomically distinct source locations that differentially modulate the somatic response (Binzegger et al., 2004; Lafourcade et al., 2022; Shepherd, 2004). This spatial separation of distinct classes of inputs explains how a single neuron (or circuit, for that matter) can play different functional roles in unification, from one time step to the next.

The translation in Figure 4 shows how to resolve the perceived mismatch between hierarchy and brain networks, going from parse trees to function composition to neural processing. When cast in functional terms, static hierarchical phrase structure trees can be given a dynamic interpretation in terms of recurrent neural processing with the appropriate memory structure. It also shows that hierarchical processing does not require the construction of explicit representations of linguistic trees and their binding relations (as some models have suggested, e.g., Martin & Doumas, 2017; Papadimitriou & Friederici, 2022; van der Velde & de Kamps, 2006) because these relations are already implicitly present in the intermediate processing outcomes of the state-dependent neural parser. As words are being processed one by one, the system incrementally computes an interpretation in neuronal memory registers, i.e., dynamical variables in processing memory, which are a particular substate of the complete system state. Parsing “the cat chases a dog” versus “a dog chases the cat” results in distinct trajectories whose end-states represent different meanings. This procedure is analogous to evaluating a hierarchically structured arithmetic expression by a compiled program where the final outcome is a number that corresponds to the correct interpretation, rather than an explicit structural representation of the binary expression tree. Introspection of constituent structure requires linguistic knowledge and should not be considered part of automatic language processing.

Function composition and binding in comprehension rely on data structures that must be supported by neurobiology. The nature of these data structures determines the kind of unification procedures that can run on partial interpretations temporarily held in processing memory. Data structures and how they are represented in memory are a key organizing principle of neurobiological information processing systems. For example, the membrane potential, or other dynamical variables, of a biological neuron assumes real number values. The decimal expansion of these numbers can naturally be interpreted as a stack memory when combined with push and pop operations. These operations can be implemented through multiplication that shifts decimals into (push) or out of (pop) the decimal expansion, and there is evidence that single neurons can accomplish this (Groschner et al., 2022). More broadly, scaling and other operations on dynamical variables can be viewed as generalized push and pop operations.

In classical computability theory (Cutland, 1980), binding is achieved in that variables are physical memory addresses and the stored bit patterns are their current values. Composite data structures are then assembled by computing references to existing memory content, e.g., using pointers. However, since recurrent networks are fully equivalent to the classical notion of computation (Siegelmann, 1999), binding can also be achieved by neural networks. Binding is therefore not a fundamental barrier, and it is an empirical question how it is realized within the specific neurobiological memory architecture. For instance, similar to memory in digital computers, any dynamical variable in physiology with a non-zero time constant is stateful and can act as a memory register. Different information sources can be bound in these registers through temporal integration. Whether this form of binding is sufficient to explain language comprehension or whether other complex neurobiological data structures are required is an open issue, and causal models together with experimental work are needed to answer this question.

## OUTLINE OF A CAUSAL LANGUAGE MODEL

**Figure 5.**
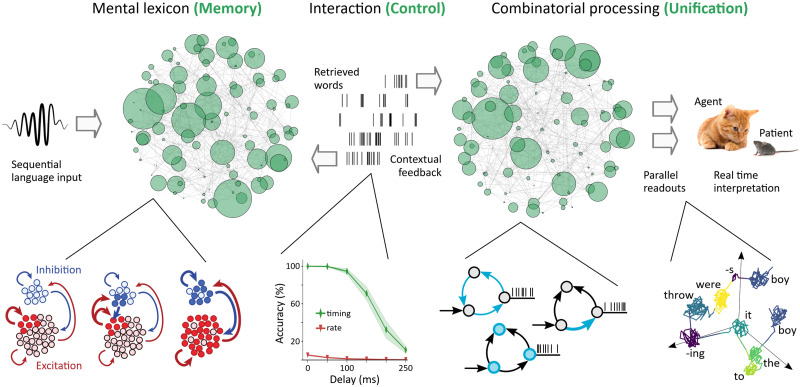
Core computational machinery for language processing. The cognitive architecture for language consists of a mental lexicon for the encoding, maintenance, and retrieval of words and a unification network for combinatorial processing. Both components require memory on long and short time scales to different degrees, and their interaction is a form of reciprocal control. Downstream readouts project the neural states of unification onto a semantic interpretation in real time. The distinction between memory, unification, and control is purely functional; it is not a claim about anatomical localizability. Any computational system, whether neural or classical, implements these components in one way or another. Insets from left to right: word representations in the mental lexicon, or engrams, are strongly coupled cell assemblies, recruiting excitatory or inhibitory synapses, or both (bold arrows); figure adapted from Hennequin et al. (2017). Retrieved words are encoded as spike trains that drive unification, and information content is better preserved in the timing of spikes than in spike rates, even in the presence of noise; figure adapted from Uhlmann (2020). Processing memory for unification may be implemented neurobiologically as network attractors, short-lived synaptic facilitation, or intracellular adaptation that transiently changes neuronal excitability (top to bottom). History-dependent processing in unification, where the current state is folded together with incoming input, separates multiple occurrences of the same word (here “boy”) in neural state space, and this can be used to establish binding relations between words and their semantic roles; figure adapted from Fitz et al. (2020).

Unification instantiates a generic sequence processor that may not be specific to language (Jackendoff & Audring, 2019; Petersson & Hagoort, 2012) and establishes semantic relations between constituents (e.g., *who does what to whom*?) within processing memory (Figure 5). Traditionally, neurobiological short-term memory has been conceptualized as states of persistent neural activity (Fuster & Alexander, 1971; Goldman-Rakic, 1995). Persistent activity can be achieved through cellular bistability (Loewenstein & Sompolinsky, 2003; Zylberberg & Strowbridge, 2017) or attractor dynamics where excitatory feedback enables the replay of information beyond stimulus offset (Barak & Tsodyks, 2014; Durstewitz et al., 2000). Alternatively, short-term memory has been linked to functional connectivity induced by transient changes in synaptic efficacy (Fiebig & Lansner, 2017; Mongillo et al., 2008). These theories can explain maintenance and cued recall, but they have not been developed with language in mind. A neurobiological processing memory for language also needs to be able to integrate and transform internal representations in an online, incremental fashion and actively compute an interpretation from rapid serial input. In addition, this memory system needs to be context-dependent and sensitive to precedence relations between words. Recent modeling work indicates that these requirements are met by neuronal processing memory (Fitz et al., 2020; Rao et al., 2022), which is grounded in the observation that neurons exhibit adaptive changes in excitability as a function of experience (Marder et al., 1996; Turrigiano et al., 1996). This intrinsic plasticity is common in excitatory cortical cells (Gouwens et al., 2019), and adaptive changes can last from milliseconds (Koch, 1998) to seconds (Levy & Bargmann, 2020) to minutes (Titley et al., 2017). Network simulations have shown that neuronal memory can support sentence-level semantic processing and that memory span was proportional to the time constant of spike-rate adaptation (Figure 5). The proposed memory mechanism was also suitable to resolve temporary ambiguity and establish binding relations between words and their semantic roles when queried (Fitz et al., 2020; Uhlmann, 2020). It is likely that other factors contribute to neuronal memory as well, including the kinetics of NMDA-receptors (Lisman et al., 1998) and the morphology of dendrites (Papoutsi et al., 2014; Poirazi & Papoutsi, 2020). These two features support the generation of plateau potentials, endowing neurons with dendritic memory that is useful for structured sequence processing on short time scales (Quaresima et al., 2023). These findings from causal modeling illustrate how evidence from neurobiology can generate new hypotheses about the nature of processing memory for language. Noncausal models do not express these cellular and synaptic features and might therefore miss crucial neurobiological memory mechanisms.

Storage in the mental lexicon requires persistent adaptation on longer time scales than unification. Engrams in long-term memory are viewed as strongly connected cell assemblies that encode information into synaptic conductances through spike-timing dependent plasticity (Caporale & Dan, 2008; Miehl et al., 2023; Poo et al., 2016; but see Gallistel, 2021). There is less consensus, however, on whether engrams are exclusively located in excitatory synapses or also involve inhibitory ones (Hennequin et al., 2017), perhaps even primarily (Mongillo et al., 2018). Previous work has shown that engrams can emerge from sparse, random networks when multiple mechanisms for unsupervised learning and homeostatic regulation interact dynamically (Litwin-Kumar & Doiron, 2014; Zenke et al., 2015). In these simulations, acquired memories were relatively stable in the presence of background noise and ongoing plasticity and could be reactivated reliably after delay. These causal models of long-term storage can serve as a starting point for a neurobiological model of the mental lexicon. A promising first step in this direction has been taken in Tomasello et al. (2018).

Words in the mental lexicon have a feature structure consisting of, among others, semantic, syntactic, and morphological attributes that are stored and maintained in the neurobiological infrastructure of the brain. Words can also include larger units such as, e.g., collocations, multiword expressions, idioms, or argument-structure constructions. In retrieval, speech sounds or letter sequences have to be recognized as particular words while, concurrently, these features are being computed from partial cues (pattern completion). Hence, there are at least two computational tasks that need to be solved in lexical retrieval; they happen in parallel and are likely to interact. For example, word recognition itself might sharpen the selection of features activated prior to the recognition point, perhaps through lateral inhibition. The computation of lexical features is currently not addressed by existing models that have focused on recognition only (e.g., those reviewed in Hannagan et al., 2013; Magnuson et al., 2020; Weber & Scharenborg, 2012). A causal model of the mental lexicon is needed that can explain how words are represented within the neurobiological substrate and how their feature structure is “activated” from perceptual input (Poeppel & Idsardi, 2022). Furthermore, the mental lexicon is language-specific, rapidly acquired in development through local learning mechanisms, and uniquely human. To explain these traits in neurobiological terms is another important challenge for causal modeling (see Box 3 in the Concluding Remarks).

The mental lexicon and unification continuously interact through feedback loops and exert reciprocal control (Figure 5). The feature structure of retrieved words controls the combinatorial operations of the unification network, and, conversely, the partial interpretations computed by unification control the context-dependent retrieval process when multiple candidates are compatible with the sensory signal. To develop a combined architecture for adult language processing, the synaptic pathways for information exchange between these different functional modules can be fine-tuned using methods from control theory (Kao & Hennequin, 2019), feedback learning (Nicola & Clopath, 2017), or error-based optimization of networks (Neftci et al., 2019).

Compared to other cognitive domains, causal language modeling is in a privileged position because linguistic theory/analysis provides an extensive list of conceptual primitives that form the elementary units of language (which has been referred to as the “parts list”; Poeppel, 2012). In addition, a basic functional architecture can be derived from findings in cognitive neuroscience and the theory of computation (Figure 5). Hence, causal language modeling can draw on a rich set of reference points across Marr’s (1982) descriptive hierarchy; we know, roughly, which units and procedures to look for in neurobiology. However, if conceptual primitives and computational routines cannot be explicated in neurobiological terms, their theoretical status may eventually have to be revised.

## MODELS OF BEHAVIOR VERSUS THE SYSTEM

Computational language models that operate at the algorithmic level are often tested against linguistic behavior, i.e., system output or data collected in some experiment. The better a model reproduces or predicts behavior, the better it is considered to be validated. There are other adequacy criteria as well, but behavioral fit is a primary source of evidence in cognitive modeling. Causal models, on the other hand, are mainly concerned with the neurobiological mechanisms of the underlying system. They aim to be explanatory at the level of implementational causality: How do inputs give rise to outputs within the neurobiological machinery for language? Causal models are therefore not primarily about observations or behavior but—in the first instance—about the mechanisms that generate behavior. The extent to which the behavior of a causal model is human-like is determined by the degree to which it approximates the biophysical characteristics of the actual system; fit with behavioral data is an independent outcome and not the immediate modeling goal (Box 2). This approach is reminiscent of early connectionist language models, which also intended to derive behavior from principles of neural information processing (e.g., Elman, 1990; McClelland et al., 1989). Today, it is widely held that these models incorporate too little neurobiological detail (see Figure 2) to be viewed as causal models of the neurobiological system (Arbib et al., 2000; Craver, 2006; Karaminis & Thomas, 2012). Deep learning approaches to natural language processing (see Young et al., 2018, for an overview), which are an extension of the connectionist paradigm, are very powerful in generating language-like output and might be a useful heuristic. However, large language models (LLMs) are neither models of human behavior nor models of the neurobiological machinery. They do not model the causal structure of the language system nor cognitive function as such (language comprehension differs from next-word prediction; Bender & Koller, 2020), and they are sometimes inadequate behaviorally in that they fail in nonhuman ways and do not fail in human ways (Marcus, 2018; but see Linzen & Baroni, 2021, for a different perspective). Using LLMs to fit brain data (e.g., Goldstein et al., 2022; Schrimpf et al., 2021) is correlational rather than causal in nature. Hence, it is debatable whether they contribute novel insights to the study of human language at the implementational level of Marr’s hierarchy.

Box 2. Methodological road map.
Causal modeling initially puts priority on neurobiological realism over fit with behavioral data. Therefore, a first step is to create models from neurobiological components parts that can accomplish core computational tasks involved in language processing at the algorithmic level. Network features should comply with functionally relevant neurophysiological measurements. These include the electrophysiological properties of different neuron types (e.g., their resting potential, rheobase, membrane time constant, spike threshold, etc., that can be obtained from databases such as NeuroElectro (Tripathy et al., 2014) and the Allen Brain Map), spontaneous and evoked firing rates (Attwell & Laughlin, 2001; Roxin et al., 2011), the quantized range of synaptic conductances (Bartol et al., 2015), the ratio of excitation and inhibition (Abeles, 1991; Xue et al., 2014), and the distribution of major receptor types across regions in the language network (Zilles et al., 2015; Duarte et al., 2017). Language models with these characteristics have face validity since they are grounded in experimental neurobiology. This approach applies equally to networks of any spatial scale, including larger-scale neocortical or cortico-striatal networks (Haber, 2016; Mountcastle, 1997; Shepherd, 2004). In each case, the connectivity matrix would be structured into blocks with specified neuron types and local connectivity as well as specific between-region connectivity (cf. also Pulvermüller et al., 2021).

Key language tasks include, among others, the transduction of auditory signals onto equivalence classes (phonemes), the retrieval of lexical features (semantic, morphosyntactic, etc.) from these units of speech, and the integration of recognized words into a sentence-level interpretation (semantic dependency structure). To gauge task performance, simple parallel readout classifiers can be used as a measurement device that maps nonlinear circuit activity onto linguistic categories (Buzsáki, 2010; Rigotti et al., 2013). Thus, readouts are a diagnostic tool to probe whether a given dynamical system can be harnessed to compute linguistic functions. The neurobiological features of this system can then be manipulated (another meaning of *causal* modeling) and their computational contribution can be determined through model comparisons as a method of investigation (Duarte & Morrison, 2019; Fitz et al., 2020; Uhlmann, 2020). Importantly, failure to achieve these language tasks is inherently meaningful because it points directly to missing neurobiological features that might be important for language processing. In addition, our current best models of neurobiological components may have to be refined or extended in light of new empirical evidence while the causal modeling framework does not need to be questioned as such.

Once a basic neurobiological language model has been established, causal modeling can begin to bridge into empirical data and linguistic behavior. For instance, local field potentials can be synthesized from perisynaptic activity in simulated spiking networks (Hagen et al., 2016; Mazzoni et al., 2015) to connect causal models to ECoG, EEG, and MEG data. In similar vein, hemodynamic response models have been proposed to link in silico network activity to fMRI data (Bonaiuto & Arbib, 2014). These methods can be used to relate causal models to functional neuroimaging. This endeavour also involves statistical approaches to quantifying single-neuron and population dynamics (Kass et al., 2018; Saxena & Cunningham, 2019) and the representational analysis of biological networks (Barrett et al., 2019). Novel techniques for analytic synthesis need to be developed that allow the abstraction of adaptive dynamical systems to discretized combinatorial models.

Causal modeling advances from neurobiological models of algorithmic capacities to neuroimaging data and linguistic behavior. Through incremental model refinement, the core objective is to uncover the computational role of neurobiological features and synthesize a computational neurobiology of language across levels of explanation.

In models of behavior, variables and parameters are dimensionless scalars that do not correspond to measurable quantities in biological reality and often lack interpretability in cognitive terms (Eckstein et al., 2022). In causal models, they have physical units of measurement (e.g., mV, nS, pF) that need to fall within physiological bounds. This restricts parameter choices to empirical ranges, reduces degrees of freedom, and puts strong constraints on the model space M (see Figure 3). Since units have to match on both sides of dynamical equations, causal models are also internally consistent. Whereas cognitive models often attempt to capture behavior with as few parameters as possible, the challenge for causal modeling is to deal with the abundance of parameters provided by the neurobiological system (e.g., on the order of ≈10^14^ synaptic conductances). We refer to this distinction as the statistician’s versus the neuroscientific perspective. Consequently, standard model selection criteria do not apply in causal modeling (e.g., Occam’s razor). What needs to be explained is how the neurobiological language system can generalize appropriately despite being nominally overparameterized (Hasson et al., 2020). A third difference concerns the relationship between model time and real physical time. In cognitive models of behavior, time is often expressed in terms of processing steps and the relation to physical time is typically arbitrary. In causal models, time corresponds to real physical time since it arises from the dynamics of neuronal integration and synaptic transmission (Gerstner et al., 2014). Due to this inherent correspondence, a causal model would allow us, in principle, to investigate how speech and language processing unfold in time at any desired resolution. More importantly, however, causal models are therefore strongly constrained by real-time processing requirements, whereas models of behavior typically are not.

Another difference between models of behavior and causal models of the system is related to their explanatory status. Output or behavior of a system should not be mistaken for the mechanisms that generate behavior at the level of physical, or neurobiological, causes. For example, a statistical model of weather data can have high predictive accuracy, but it is not a model of Earth’s atmosphere that generates the weather. Similarly, no one would confuse a regression model of experimental data with a model of the processes that generated the data. By parity of reasoning, suppose a cognitive language model reproduces all known behavioral data. This would not guarantee that the model correctly describes the algorithms employed by the brain, and it would still be unclear whether the model is explanatory with respect to the causal generators of behavior. This uncertainty persists until it has been demonstrated that a proposed algorithmic model can be reduced to the relevant neurobiology. Similar uncertainty afflicts experimental approaches that attempt to reverse engineer the computational machinery for language from behavioral output. Neuroimaging methods (fMRI, EEG, MEG, etc.) observe sequences of brain states, i.e., processing outcomes or system behavior in the broadest sense, but not the neurobiological processing dynamics itself, which is hidden from the measurement devices. Another complication is that the fMRI signal, for example, is related to the blood oxygen level dependent response which in turn is related indirectly to neural activity. Language models that are inferred from such data are confounded by these theoretical linking principles, which need to be factored out in order to arrive at a veridical model of the neurobiological processing machinery.

Simulations of the language system at the level of implementational causality are not confounded in this way and enable us to study candidate processing dynamics with unrivaled spatio-temporal precision. Moreover, component parts that lack neurobiological support do not enter into model design to begin with. Reduction has already been achieved at the level of computational elements and their interaction. Hence, neurobiological causal models describe the mechanistic generators of linguistic behavior from which observed behavior can be derived. Without a neurobiological foundation, modeling behavior is not explanatory with respect to the causal generators of behavior unless such models can be shown to be reducible to neurobiology. It is understood that a causal modeling approach requires a long-term perspective; it will take time and effort for it to succeed.

## CAUSALITY, REDUCTION AND ABSTRACTION

David Marr (1982) considered the functional level to be the most important one for understanding biological information processing systems but emphasized that different questions need to be addressed at different levels of description. He also pointed out that the different levels are “logically and causally related” (p. 25). In particular, the algorithmic level is not autonomous with respect to the implementational level. Among a number of candidate language models, it is neurobiology that is going to select the correct one, if any. There might be multiple abstractions that are equivalent in some deep sense, but there is still a matter of fact in the brain which of these abstractions is valid. For instance, recursive function theory itself can be formulated within many different mathematical frameworks, but it is an empirical question which algorithmic model the brain implements to “run” this theory. Surely, the brain does not implement language like Conway’s *Game of Life* or *Baba Is You*, both of which are Turing universal (Rendell, 2002; Su, 2023). Thus, although methodologically any of Marr’s levels can serve as a starting point, neurobiology is ontologically prior since it determines the algorithms that are implemented by the real system which, ultimately, also determine the range of possible language behavior we can observe. Both algorithm and behavior are caused by the underlying neurobiology, while the converse is not true.

In light of these dependencies, we should therefore not be satisfied to describe language at a single level only; the ambition must be to link and traverse levels through explanatory bridging principles. As an analogy, a structured computer architecture with its many layers of abstraction can be used through an operating system because the interfaces between layers are correctly designed (Tanenbaum & Austin, 2013). In other words, a higher level of abstraction has to comply with and systematically relate to lower level mechanisms by reduction. Thus, it is only under the condition of reducibility that we can “ignore” lower levels. What is currently underspecified in cognitive theories of language are precisely these interfaces between levels of abstraction. Despite decades of computational work it has not been possible to connect cognitive language models to neurobiology in a substantial manner. With a few notable exceptions (Fitz et al., 2020; Rolls & Deco, 2015; Tomasello et al., 2018), models of language processing that are characterized as neurocomputational or neurally plausible do not yet make sufficient contact with the basic neurobiological principles described in Figure 2. This also holds for language models in deep learning. The assumption that we can abstract away from these principles needs to be scientifically justified because abstraction without reduction is likely to result in simplifications that may not be valid.

Within the computer metaphor, the terminology of cognitive theory is comparable to a high-level programming language, like Python or Julia. Underneath this layer of abstraction lies the hardware-dependent machine language of the implementational substrate. The machine language determines the basic set of instructions, data types and memory registers that are instantiated by the actual neurobiological system. This instruction set architecture (ISA) corresponds to circuits built from biological neurons, their membrane potentials, spike-generation mechanisms, synaptic currents, dendritic integration, etc. A cognitive theory of language that is empirically adequate must be realizable in this neurobiological ISA, otherwise it remains disconnected from the implementational level of description. Causal models, on the other hand, are directly formulated in the language of the neurobiological ISA and pinpoint the fundamental computational elements in neurobiology, their interactions, and how they support language functions.

Although causal models describe language processing in terms of biological neurons and synapses, one long-term goal is to abstract a homomorphic cognitive model from the neurobiological specification that instantiates a correct algorithmic description of the language faculty. There is, of course, no guarantee that any particular causal model will yield a correct cognitive theory. But any cognitive theory that is correct needs to be consistent with what is known about the language system from a neurobiological perspective. A similar point has been made by Feldman (2006), in more general terms. Through simulation, analysis, and theoretical insight, the aim is to *discover* rather than guess the algorithms that operate at the neural level. These algorithms, in addition, have to explain the breathtaking speed, fault tolerance, and energy efficiency of the brain system for language. The functionalist doctrine and multiple realizability, which are only concerned with nonbiological input–output relations, have no bearing on these issues.

Validation of causal models involves different sources of evidence, including behavior, none of which is sufficient on its own. In this sense, causal modeling is not intrinsically reductionist but aims to encompass all of Marr’s levels in the final analysis (Figure 1 and Box 2). Models that are behaviorally adequate but violate known neurobiology cannot be correct. Models that are behaviorally inadequate but consistent with known neurobiology need to be refined. Thus, causal modeling advocates an iterative approach that seeks to gradually approximate language behavior from first principles of neurobiology, through cycles of model development, validation, and revision.

## CONCLUDING REMARKS

The nature of the language faculty—its representations, storage mechanisms, and elementary operations—is determined by the neurobiological infrastructure that sustains it. A large number of replicable findings from experimental neuroscience (Luo, 2015; Sterling & Laughlin, 2015) have been formalized as effective mathematical models (Gerstner et al., 2014) that can readily be used as the basic building blocks for causal language modeling. Complex systems assembled from these neurobiological component parts are analytically intractable, and simulation therefore becomes a methodological necessity (Einevoll et al., 2019; Gerstner et al., 2012). With unprecedented access to computational power and neurobiological insight to constrain these simulations, it is the appropriate time to supplement traditional methods in language research with causal modeling in order to integrate language across levels of description. Neurobiological causal modeling follows the classical path of science in attempting to understand complex systems—e.g., multicellular organisms, condensed matter, or planetary climate—from observations to statistical modeling to explaining the causal structure of the physical system that generated the observations in the first place. Eventually, this approach might even allow us to bridge into the genetic basis of language.

Box 3. Open questions.

- What are the elementary units of language in neurobiological terms (e.g., phonemes, syllables, words, phrases, clauses, semantic roles, event structure)? Which neural data structures encode these units and their composition, and how can these data structures be identified through causal modeling?
- What is the functional role of brain structure in language processing across spatial scales, including structure in the dendritic tree of neurons, laminar structure in cortical microcircuits, and connectivity structure between brain regions in the perisylvian language network?
- What is the neurobiological correlate of processing memory for unification? How does this system support temporal integration, the resolution of non-adjacent dependencies, and recursive function calls for compositional processing? How are intermediate processing outcomes stored, retrieved at the right point in time, and broadcast to where they will be used next?
- How is prior knowledge of language expressed within the neurobiological infrastructure of the language-ready brain and what is unique about human neurobiology that enables language in the first place? Causal modeling is ideally suited to test specific hypotheses concerning, e.g., dendritic morphology, cytoarchitectonic composition, receptor-architectonic fingerprints, and anatomical connectivity.
- What is the structure of words stored in the mental lexicon and how does it enable combinatorial sentence-level processing in biological networks? What kinds of representations are supported by the underlying neurobiology? How are they encoded and maintained in long-term memory in the presence of noise and ongoing plasticity, and how is the feature structure of words computed from partial cues?
- How is a language-specific mental lexicon acquired given the weak, local neurophysiological learning mechanisms currently known, and how does learning interact with innate structure during acquisition?
- The complexity of neurophysiology demands reduced mathematical models that abstract away from, e.g., ion channels and the molecular machinery of synapses. What is the appropriate level of reduction that is computationally feasible while still being informative at the algorithmic level?

Computational models of cognitive function are in need of stronger neurobiological foundations (O’Reilly, 2006) and several recent perspective articles have similarly suggested to “close the mechanistic gap” (Paquola et al., 2022) by means of neurobiologically grounded models of information processing (Pulvermüller et al., 2021). Our proposal is focusing on the language domain where computational models have played a particularly prominent role. However, neurobiological causal modeling amounts to more than neural network modeling with a few added constraints. Rather, we propose to reconceptualize computational language modeling and start building causal models from the ground up. This approach will not only address the missing interfaces between levels of description but is also expected to have profound ramifications at the algorithmic and functional levels themselves (Larkum, 2022). We call to action the community of language researchers to engage with this complementary approach and confront the challenges of investigating the neurobiology of language on the basis of first principles of brain organization. A joint, multidisciplinary effort is needed to bring this research program to fruition.

Causal models are formulated as systems of coupled differential equations, which is the lingua franca of science. They describe the fundamental dynamical principles underlying cognitive function in neurobiology. Hence, they provide a common, unified framework for modeling cognition that makes different instantiations of causal models commensurable and falsifiable (Haeffel, 2022; Popper, 1959). In the long term, this approach will lead to better theories of language processing, the progressive accumulation of scientific knowledge, and a deeper understanding not only of language but also other cognitive phenomena.

## ACKNOWLEDGMENTS

We would like to thank three anonymous reviewers for their helpful comments and discussion. Also, we would like to thank C. Randy Gallistel and Alessio Quaresima for their valuable feedback on earlier drafts of the manuscript.

## FUNDING INFORMATION

Netherlands Organisation for Scientific Research (NWO), Award ID: 024.001.006.

## AUTHOR CONTRIBUTIONS

**Hartmut Fitz**: Conceptualization: Equal; Writing – original draft: Lead. **Peter Hagoort**: Funding acquisition: Lead; Writing – review & editing: Lead. **Karl Magnus Petersson**: Conceptualization: Lead; Writing – original draft: Equal.

## TECHNICAL TERMS

Time scale: A characteristic time span within which a particular change in a dynamical variable takes place.
Laminar structure: Neocortical organization into six layers with characteristic connectivity within and between layers that forms cortical microcircuits.
Synaptic motif: Synaptic connectivity pattern involving a small number of neurons, e.g., the bidirectional coupling between excitatory cells, or feed-forward inhibition.
Action potential: Brief electrical pulse (spike), with a generic shape and duration (1–2 ms) generated at the cell body when a threshold is exceeded. It propagates down the axon, which connects to the dendrites of other neurons through one or more synapses.
Dendrite: Branched, tree-like structure protruding from the cell body of a neuron that integrates synaptic input to change the neuron’s membrane potential.
Point neuron: Mathematical model of a biological neuron that lumps all neuronal structure into a single, homogeneous compartment that receives input signals and generates output spikes.
Unsupervised learning: Innate, self-organized form of learning that detects patterns in unlabeled input without explicit instruction.
Homeostasis: When physiological variables deviate from a pre-set range of values, self-correcting feedback restores a dynamic equilibrium to retain stability.
Conductance: The ease with which an electric current flows through an object or material; the inverse of resistance.
Monoamine: Class of neurotransmitters that alter the processing characteristics of entire circuits beyond the single synapse; e.g., dopamine, serotonin, histamine.
Neuropeptide: Signaling molecule, or chemical messenger, that diffuses over broad areas and modulates neural activity.
G protein: Protein that transmits signals from the exterior to the interior of a cell, acting as a molecular switch.
Receptor: Transmembrane protein that is activated by a neurotransmitter and regulates the activity of synaptic ion channels across the cell membrane.
Poisson process: A stochastic event process where random events are independent of each other and the time between events follows an exponential distribution.
Neurotransmitter: Molecule that transmits signals across a chemical synapse from one neuron to another, e.g., glutamate or *γ*-aminobutyric acid (GABA).
Dynamical variable: A physical quantity whose numerical value changes over time, describing some aspect of the system’s state, e.g., the membrane potential of a neuron, or the conductance of a particular synapse.
Engram: A basic unit of information stored in long-term memory, e.g., a phoneme, word, or idiomatic expression.
Spike-timing dependent plasticity: Adaptive mechanism that adjusts the strength of synapses based on the relative timing of a neuron’s input and output spikes, leading to, e.g., long-term synaptic potentiation or depression.
Rheobase: The minimal current amplitude of infinite duration that results in the discharge of an action potential.
